# Dominant resistance to Bt cotton and minor cross-resistance to Bt toxin Cry2Ab in cotton bollworm from China

**DOI:** 10.1111/eva.12099

**Published:** 2013-09-17

**Authors:** Lin Jin, Yiyun Wei, Lei Zhang, Yihua Yang, Bruce E Tabashnik, Yidong Wu

**Affiliations:** 1 Key Laboratory of Integrated Management of Crop Diseases and Pests (Ministry of Education), College of Plant Protection, Nanjing Agricultural UniversityNanjing, China; 2 Department of Entomology, University of ArizonaTucson, AZ, USA

**Keywords:** dominant resistance, resistance evolution, resistance management

## Abstract

Evolution of resistance by insect pests threatens the long-term benefits of transgenic crops that produce insecticidal proteins from *Bacillus thuringiensis* (Bt). Previous work has detected increases in the frequency of resistance to Bt toxin Cry1Ac in populations of cotton bollworm, *Helicoverpa armigera*, from northern China where Bt cotton producing Cry1Ac has been grown extensively for more than a decade. Confirming that trend, we report evidence from 2011 showing that the percentage of individuals resistant to a diagnostic concentration of Cry1Ac was significantly higher in two populations from different provinces of northern China (1.4% and 2.3%) compared with previously tested susceptible field populations (0%). We isolated two resistant strains: one from each of the two field-selected populations. Relative to a susceptible strain, the two strains had 460- and 1200-fold resistance to Cry1Ac, respectively. Both strains had dominant resistance to a diagnostic concentration of Cry1Ac in diet and to Bt cotton leaves containing Cry1Ac. Both strains had low, but significant cross-resistance to Cry2Ab (4.2- and 5.9-fold), which is used widely as the second toxin in two-toxin Bt cotton. Compared with resistance in other strains of *H. armigera*, the resistance in the two strains characterized here may be especially difficult to suppress.

## Introduction

The insecticidal proteins of *Bacillus thuringiensis* (Bt) kill some major insect pests, but are harmless to vertebrates and most other organisms (Mendelsohn et al. 2003; Sanahuja et al. 2011; Pardo-López et al. 2013). Corn and cotton plants genetically engineered to produce Bt toxins have provided many benefits including pest suppression, reduced use of insecticide sprays, conservation of natural enemies, increased yield, and higher farmer profits (Wu et al. 2008; Carpenter 2010; Hutchison et al. 2010; National Research Council 2010; Tabashnik et al. 2010; Edgerton 2012; Kathage and Qaim 2012; Lu et al. 2012). Since 1996, farmers worldwide have planted transgenic crops producing Bt toxins on a cumulative total of more than 480 million ha, including 70 million hectares in 2012 (James 2012). The most widely used Bt proteins are crystalline (Cry) toxins, particularly three toxins that kill lepidopteran larvae: Cry1Ab in Bt corn, Cry1Ac in Bt cotton, and Cry2Ab in second-generation Bt corn and Bt cotton (Tabashnik et al. 2009b).

The primary threat to the continued efficacy of Bt toxins is evolution of resistance by pests (Tabashnik 1994; Gould 1998; Ferré and Van Rie 2002; Tabashnik et al. 2009b, 2013). Field-evolved (or field-selected) resistance is defined as a genetically based decrease in susceptibility of a population to a toxin caused by exposure of the population to the toxin in the field (Tabashnik et al. 2009b). Field-evolved resistance associated with reduced efficacy of Bt toxins has been reported in some populations of seven pest species: two targeted by Bt sprays (Tabashnik et al. 1990; Janmaat and Myers 2003) and five targeted by Bt crops (Luttrell et al. 2004; Van Rensburg 2007; Tabashnik et al. 2008, 2013; Storer et al. 2010; Dhurua and Gujar 2011; Gassmann et al. 2011). Other cases of significant decreases in susceptibility to the Bt toxins in transgenic crops including ‘incipient resistance’ and ‘early warning’ of resistance have been detected in at least four additional pest species (Downes et al. 2010; Alcantara et al. 2011; Zhang et al. 2011; Huang et al. 2012; Wan et al. 2012).

In particular, increases in the frequency of resistance to Cry1Ac have been reported in populations of the major cotton pest, cotton bollworm (*Helicoverpa armigera*), from northern China, where Bt cotton that produces Cry1Ac has been grown intensively for more than a decade (Liu et al. 2010; Zhang et al. 2011, 2012a). Decreased susceptibility to Cry1Ac in populations of *H. armigera* from China has been documented with monitoring data from a number of studies based on comparisons over time within populations exposed intensively to Bt cotton and between populations that differ in their history of exposure to Bt cotton (Wu et al. 1999; Li et al. 2007, 2010; Yang et al. 2007; An et al. 2010; Liu et al. 2010; Zhang et al. 2011, 2012a). Nonetheless, the maximum percentage of resistant individuals reported in a population is 2.6% (compared with 0% for susceptible populations), and Bt cotton producing Cry1Ac has continued to provide substantial control of this pest in China (Zhang et al. 2011). Thus, the small but statistically significant increases in the frequency of resistance noted above provide an early warning of resistance that could become a more serious problem (Zhang et al. 2011).

The main strategy for delaying evolution of pest resistance to Bt crops relies on refuges of host plants that do not produce Bt toxins, which promotes survival of pests susceptible to Bt toxins (Gould 1998; Tabashnik et al. 2004). Ideally, most of the rare resistant pests surviving on Bt crops will mate with the relatively abundant susceptible pests from nearby refuges. If inheritance of resistance is recessive, the progeny from such matings will die on Bt crops, substantially delaying the evolution of resistance. Conversely, if inheritance of resistance is dominant, the progeny from matings between resistant and susceptible adults will survive on Bt crops, and refuges will be less effective for delaying resistance.

The refuge strategy has been used with first generation Bt plants that produce a single Bt toxin and with more recently introduced Bt crop ‘pyramids’ that produce two or more Bt toxins that kill a given pest (Zhao et al. 2005; Brévault et al. 2013). Pyramids are expected to be most effective for delaying evolution of resistance if adaptation to one toxin in the pyramid does not cause cross-resistance to the other toxin(s) in the pyramid (Zhao et al. 2005; Tabashnik et al. 2009a; Brévault et al. 2013). Whereas Bt cotton producing only Cry1Ac is grown in China, second-generation Bt cotton plants producing toxins Cry1Ac and Cry2Ab are grown in Australia, India, and the United States (Tabashnik et al. 2013).

Because the refuge and pyramid strategies for delaying resistance require understanding of the dominance of resistance and cross-resistance, we evaluated these traits in two resistant strains of *H. armigera* isolated from populations in two provinces of northern China where Bt cotton has been grown extensively for more than a decade. We quantified dominance of resistance with the parameter *h*, which varies from 0 for completely recessive resistance to 1 for completely dominant resistance (Liu and Tabashnik 1997). Whereas *h* for resistance to a diagnostic concentration of Cry1Ac ranged from 0 to 0.66 (mean = 0.19) in 14 previously studied strains of *H. armigera* (Kaur and Dilawari 2011; Zhang et al. 2012a,2012b), it was 1 for both strains analyzed here, indicating completely dominant resistance. Also in contrast to previous results for *H. armigera* (Akhurst et al. 2003; Xu et al. 2005; Luo et al. 2007; Liang et al. 2008; Yang et al. 2009; Zhang et al. 2012b), both resistant strains had minor but significant cross-resistance to Cry2Ab.

## Materials and methods

### Bt toxins, diet bioassays, and rearing

Dr. Marianne P. Carey (Case Western Reserve University, USA) provided Cry1A activated toxins (Cry1Aa, Cry1Ab, and Cry1Ac). Cry2Ab protoxin was provided by the Institute of Plant Protection, Chinese Academy of Agricultural Sciences (CAAS), China.

We used diet surface overlay bioassays (Zhang et al. 2011) in which toxin stock suspensions were diluted with a 0.01 m, pH 7.4, phosphate-buffered solution (PBS). Liquid artificial diet (900 μL) was dispensed into each well of a 24-well plate. After the diet cooled and solidified, 100 μL of PBS containing the desired concentration of Bt toxin was applied evenly to the diet surface in each well and allowed to air dry, and a single larva was placed in each well. At the end of the bioassay, we scored larvae as dead if they died or if they weighed <5 mg.

For Cry1A toxins, we tested second instars that were starved for 4 h and we recorded mortality at 5 days, as in our previous studies (Xu et al. 2005; Yang et al. 2007; Zhang et al. 2011). For Cry2Ab, we tested unfed neonates (24 h old) and recorded mortality after 7 days, which is the method established in Australia for testing Cry2Ab against *H. armigera* (Mahon et al. 2007) and requires less toxin.

We used a diagnostic concentration of 1 μg Cry1Ac/cm^2^ diet, which in previous studies killed all larvae tested from susceptible populations (*n* > 2000), but <10% of larvae from the resistant strain SCD-r1 (Xu et al. 2005; Zhang et al. 2011, 2012a,2012b). Because this concentration kills virtually all susceptible larvae, it provides a conservative method for detecting resistance. We used a series of concentrations to estimate the concentration of each toxin killing 50% of larvae (LC_50_) for susceptible strain SCD and resistant strains AY2 and QX7 (described below). To estimate LC_50_ values, we tested 48 larvae of each strain at each toxin concentration, including a control with PBS and notoxin. We adjusted for control mortality (range = 0–8.3%) to estimate LC_50_ values, but not in the bioassays with only the diagnostic concentration, which had lower control mortality (<5%).

Larvae from all strains were reared on an artificial diet, and adults were maintained as described previously (Zhang et al. 2011). All experiments were conducted at 26°C (±1°C) and 60% (±10%) RH with 16 h L: 8 h D.

### Previously described susceptible strain SCD and resistant strain SCD-r1

The susceptible SCD strain of *H. armigera* was started with insects from the Côte d'Ivoire (Ivory Coast), Africa, over 30 years ago and was maintained in the laboratory without exposure to insecticides or Bt toxins (Yang et al. 2009). A 2010 survey showed that despite this long-term laboratory rearing, susceptibility to Cry1Ac was not greater for SCD compared with the most susceptible field populations tested (Zhang et al. 2011). The LC_50_ of Cry1Ac-activated toxin was numerically higher for SCD than for both field populations tested from northwestern China that had limited exposure to Bt cotton as well as for two of the 13 field populations tested from northern China (Zhang et al. 2011). The LC_50_ of Cry2Ab was equal to or higher for SCD compared with all of the 14 field populations tested from northern and northwestern China (Zhang et al. 2011). The recessive *r*_1_ allele of the cadherin gene (*HaCad*) was isolated from the resistant strain GYBT, which was started in August 2001 with 300 large larvae collected from Bt cotton in Gaoyang County of Hebei Province of northern China and selected with Cry1Ac for 28 generations in the laboratory (Xu et al. 2005). The resistant strain SCD-r1 was created by introgressing *r*_1_ from GYBT into SCD (Yang et al. 2009).

### Isolation of strains AY2 and QX7 with field-derived resistance

**Figure 1:**
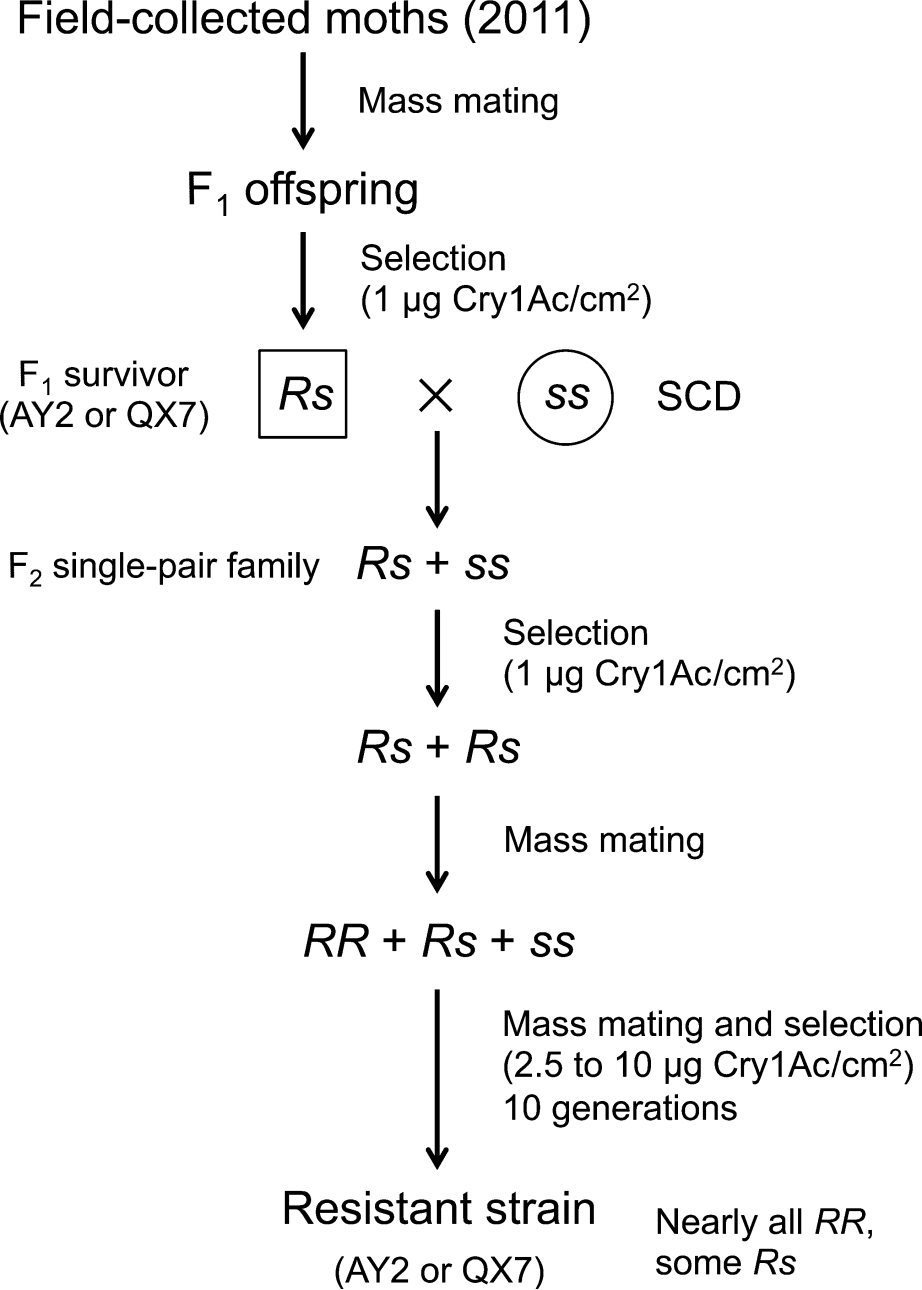
Isolation of resistant strains AY2 and QX7 from field-selected populations Anyang and Qiuxian of northern China.

Using the methods of Zhang et al. (2011), we collected *H. armigera* moths at light traps in cotton fields during June of 2011 from Anyang in Henan Province and Qiuxian in Hebei Province of northern China (Fig. 1). From each site, we pooled field-collected male and female moths (222 from Anyang, 178 from Qiuxian) and allowed them to mate (Fig. 1). The F_1_ progeny were tested at the diagnostic concentration of Cry1Ac, and survivors were reared to adults. A subset of the adult survivors from each population was paired individually with single adults of the opposite sex from the susceptible strain SCD.

We started resistant strain AY2 from a single-pair F_2_ family generated by pairing one of the resistant F_1_ males from Anyang that survived the diagnostic concentration test with one female from the susceptible strain SCD. In parallel, we started resistant strain QX7 from the single-pair F_2_ family generated by crossing one of the resistant F_1_ males from Qiuxian with one SCD female. We selected the offspring (F_2_) from each of these two single-pair crosses at the diagnostic concentration, transferred survivors to untreated diet, and allowed the surviving moths to mate among themselves within each strain. For each of the next 10 generations, we selected each strain with increasing concentrations of Cry1Ac (2.5, 5, 8, and 10 μg Cry1Ac/cm^2^ for generations 1, 2, 3–4, and 5–10, respectively). After each selection, we reared the survivors to pupation on untreated diet to continue each strain.

### Inheritance of resistance to Cry1Ac in diet

After completing the 10 generations of selection described above, we set up single-pair reciprocal crosses with one individual from a resistant strain (AY2 or QX7) and the other from the susceptible SCD strain. From 13 single-pair crosses from AY2 and 20 from QX7, we obtained F_1_ progeny and tested 48 second instars per family from each cross at the diagnostic concentration. As an internal control, we also evaluated progeny of mass crosses between resistant strain SCD-r1, which has recessive resistance (Yang et al. 2009). In all mass crosses, we used at least 30 adults of each sex. We tested for sex linkage and maternal effects by comparing results between reciprocal crosses for each strain (resistant female × susceptible male and resistant male × susceptible female). We evaluated the dominance of resistance in each of the three resistant strains by comparing survival between the resistant strain, SCD, and the F_1_ progeny from the cross between the resistant strain and SCD. We conducted backcrosses [(resistant × SCD) × SCD] and evaluated the number of loci conferring resistance with three methods: direct tests of a one-locus model, indirect tests of models with one, two, five, and ten loci, and estimation of the minimum number of effective factors influencing resistance (Lande 1981; Tabashnik et al. 1992, 2002).

### Inheritance of resistance to Bt cotton leaves in AY2 and QX7

We used methods analogous to those described above to evaluate dominance of resistance in AY2, QX7, and SCD-r1 to leaves from Bt cotton containing Cry1Ac. All larvae tested in leaf bioassays were obtained from mass crosses, including reciprocal mass crosses between each resistant strain and the susceptible SCD strain.

The leaves tested in bioassays were obtained from Bt cotton planted in the field on April 18, 2012, at Luhe in the Jiangsu Province of China. We used Bt cotton cultivar GK19, which produces a Cry1Ac/Cry1Ab fused protein (Wan et al. 2005; Tabashnik et al. 2012). GK varieties producing this protein predominate in China and accounted for 93% of China's Bt cotton in 2009 (Tabashnik et al. 2012). No insecticides were used to protect plants. We collected the top second or third leaf on the main stem on the morning of July 23, 2012, when plants were flowering. We took leaves to the laboratory in insulated boxes with ice and infested them with larvae on the afternoon of July 23, 2012.

For each replicate, we put five unfed neonates (up to 24 h old) on one cotton leaf and put the leaf into a 115-mL glass tube. To provide moisture, we inserted the petiole of each leaf into 20 mL of 1% agar at the bottom of the tube. Each tube was covered with two layers of black cloth to prevent the insects from escaping. We tested 30 replicates (150 neonates) for each of the following: SCD, AY2, QX7, SCD-r1, and the F_1_ progeny from crosses between each resistant strain and SCD. After 5 days, we scored larvae as dead if they died or if they weighed <5 mg.

### Cry1Ac concentration in field-collected Bt cotton leaves used in bioassays

A subset of the Bt cotton leaves collected from the field as described above were stored at −80°C and used later for measuring Cry1Ac concentration with an enzyme-linked immunosorbent assay (ELISA) kit (QualiPlate™ Kit; Envirologix, Portland, ME, USA). The kit is a ‘sandwich’ ELISA in which Cry1Ac binds to antibody and is detected by the addition of horseradish peroxidase-labeled antibody. For each of three replicates, we tested a leaf sample of 100 mg consisting of pieces (about 5 mg each) from 20 different leaves. We added extraction buffer (1 mL) to the tube and ground the plant tissue with a pestle. Extracts were diluted with the buffer solution at 1:200 to bring assay results within the range of calibration. To establish a standard curve, we used a microtiter plate to measure the optical density (OD) at 450 nm of a series of dilutions of Cry1Ac of known concentration. We measured the OD of leaf extracts and used the standard curve to convert OD to Cry1Ac concentration. We used this concentration to calculate the μg of Cry1Ac per gram of fresh leaf tissue.

### Data analysis

#### Probit analysis

We used the PoloPlus program (LeOra Software 2002) to conduct probit analysis of the mortality data to estimate LC_50_, the 95% fiducial limits of the LC_50_, the slope of the concentration-mortality line, and the standard error of the slope. We considered two LC_50_ values significantly different only if their 95% fiducial limits did not overlap, which is a conservative criterion (Tabashnik et al. 1987; Payton et al. 2003). We calculated the resistance ratio (RR) as the LC_50_ of a population divided by the LC_50_ of the susceptible SCD strain. We use the term ‘cross-resistance ratio’ (CRR) to refer to resistance ratio for one toxin (e.g., Cry2Ab) that results from selection with a different toxin (e.g., Cry1Ac). Our use of the LC_50_ of Cry2Ab for SCD as the divisor to calculate the CRR for Cry2Ab may underestimate cross-resistance to Cry2Ab in AY2 and QX7 because the LC_50_ of Cry2Ab was significantly higher for SCD than for six of the 14 field populations tested from northern and northwestern China (Zhang et al. 2011). For Cry2Ab, the LC_50_ was eight times higher for SCD than for the most susceptible field population, and none of the 14 field populations, including Anyang and Qiuxian, had a higher LC_50_ than SCD (Zhang et al. 2011).

#### Evaluation of dominance using bioassay data

From the data collected in this study, we calculated the dominance parameter *h*, which varies from 0 (completely recessive) to 1 (completely dominant) (Liu and Tabashnik 1997), using survival (%) at the diagnostic concentration of Cry1Ac or survival on leaves of Bt cotton as follows: *h *= (survival of F_1_ – survival of SCD)/(survival of resistant strain – survival of SCD). We also used LC_50_ values for each resistant strain, susceptible strain SCD, and their F_1_ progeny to calculate the dominance parameter *D* (Stone 1968), and we converted *D* to *h* as described by Liu and Tabashnik (1997): *h *= (*D *+* *1)/2. In addition, we analyzed data from previous studies to calculate *h* as described above. The values of *h* we calculated matched those in the papers cited with one exception: Akhurst et al. (2003) reported *h *=* *0.26, but our calculations using their reported values of LC_50_ yielded *h *=* *0.39.

#### Cross-resistance between Cry1A and Cry2A toxins in selection experiments with *H. armigera* and other lepidopteran pests

Following a previously described method (Brévault et al. 2013), we used a one-tailed Wilcoxon signed-rank test (http://www.vassarstats.net/wilcoxon.html) to determine whether the log-transformed cross-resistance ratio for Cry2A toxins was significantly greater than zero in nine Cry1Ac-resistant strains of *H. armigera*, which would indicate an overall significant cross-resistance to Cry2A toxins caused by selection with Cry1Ac. We used the same method to determine if the log-transformed cross-resistance ratio between Cry1A and Cry2A toxins was significantly greater than zero in 23 selection experiments with strains of eight species of lepidopteran pests including *H. armigera*. The Cry2A cross-resistance ratio for *H. armigera* strain LFR_10_ was originally reported as 1.014 (Luo et al. 2007), which we report here as 1.0 and consider equal to one in summarizing the data.

#### Survival at the diagnostic concentration

We used Fisher's exact test (http://graphpad.com/quickcalcs/contingency1.cfm) to determine whether the frequency of live and dead larvae from diagnostic concentration bioassays with Cry1Ac differed significantly between populations or over time for a single population. In one exceptional case (Anyang 2005 vs Anyang 2011), we used a chi-squared test with Yates’ correction because the total sample size (11 544) exceeded the algorithm's maximum for Fisher's exact test.

## Results

### Isolation of resistant strains AY2 and QX7 from field-selected populations

We isolated two strains with dominant resistance to Cry1Ac while screening *H. armigera* collected in 2011 from two field populations in northern China that had been exposed extensively to Bt cotton producing Cry1Ac: Anyang in Henan Province and Qiuxian in Hebei Province (Fig. 1). We allowed field-collected moths from each site (222 from Anyang and 178 from Qiuxian) to mate among themselves and tested the F_1_ progeny from each site at a diagnostic concentration of Cry1Ac (1 μg Cry1Ac/cm^2^ diet). Survival at the diagnostic concentration for the F_1_ offspring was 2.3% (36 of 1560) for Anyang and 1.4% for Qiuxian (24 of 1680).

We started resistant strain AY2 from a single-pair F_2_ family generated by pairing one of the resistant F_1_ males from Anyang that survived the diagnostic concentration test with one female from the susceptible strain SCD. In parallel, we started resistant strain QX7 from the single-pair F_2_ family generated by crossing one of the resistant F_1_ males from Qiuxian with one SCD female. We tested the offspring (F_2_) from each of these two single-pair crosses at the diagnostic concentration, which yielded 33% survival for AY2 and 33% survival for QX7. These results indicate that the resistant F_1_ males that started the AY2 and QX7 strains had nonrecessive resistance, because recessive resistance in these males would have yielded 0% survival in the F_2_ progeny generated by single-pair crosses with females from the susceptible strain.

In each of the next 10 generations (F_3_–F_12_), we selected larvae of AY2 and QX7 at increasing concentrations of Cry1Ac (2.5–10 μg Cry1Ac/cm^2^ diet) and reared the survivors to continue each strain. After 10 successive generations of selection, Cry1Ac resistance ratios (see Methods) were 1200 for AY2 and 460 for QX7 (Table 1).

**Table 1:** Responses to Cry1Ac of resistant (AY2 and QX7), susceptible (SCD), F_1_ (resistant × susceptible), and backcross (F_1_ × susceptible) larvae of *H. armigera*.

Source	LC_50_ (95% FL)*	Slope ± SE†	*n*	Resistance ratio‡
Strain				
AY2	50.6 (34–94)	1.1 ± 0.2	336	1200
QX7	18.5 (13–31)	1.1 ± 0.1	336	460
SCD	0.0406 (0.033–0.049)	1.9 ± 0.2	384	1.0
F_1_				
AY2 × SCD	17.6 (13–27)	1.4 ± 0.2	288	430
QX7 × SCD	6.42 (4.0–13)	1.5 ± 0.2	240	160
Backcross				
(AY2 × SCD) × SCD	1.94 (1.3–3.1)	0.84 ± 0.06	624	48
(QX7 × SCD) × SCD	1.67 (1.2–2.3)	0.94 ± 0.08	528	41

* Concentration (μg toxin/cm^2^) killing 50% of larvae and its 95% fiducial limits.

† Slope of the concentration-mortality line and its standard error.

‡ LC_50_ for a strain or progeny from a cross divided by LC_50_ for susceptible strain SCD.

### Inheritance of resistance to Cry1Ac

#### Diagnostic concentration of Cry1Ac

**Figure 2:**
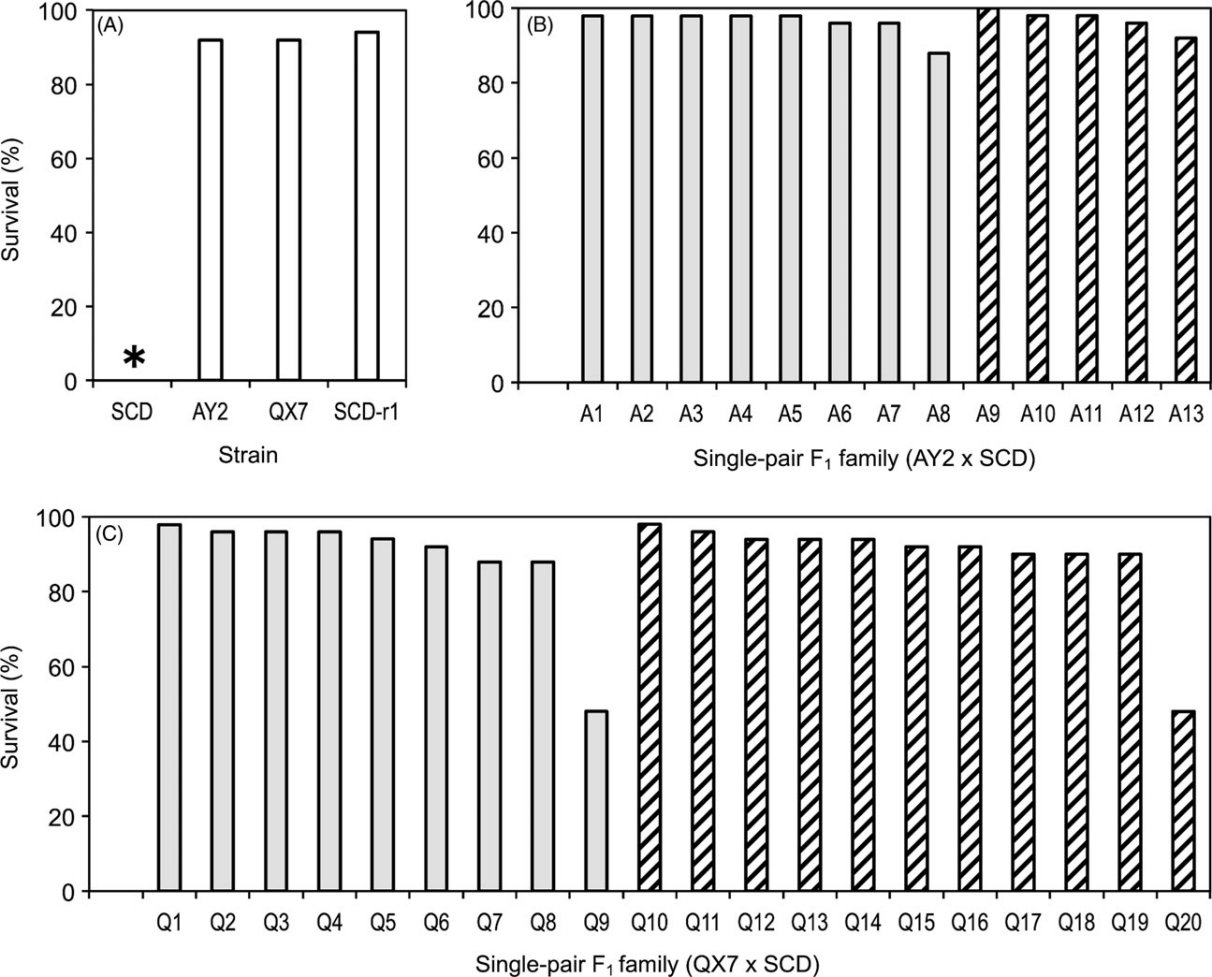
Survival of *H. armigera* larvae exposed to a diagnostic concentration of Cry1Ac (1 μg toxin/cm^2^ diet). (A) Susceptible strain (SCD) and resistant strains (AY2, QX7, and SCD-r1). The asterisk indicates 0% survival for SCD. (B) Single-pair F_1_ families from AY2 × SCD. (C) Single-pair F_1_ families from QX7 × SCD. Gray bars indicate resistant male × susceptible female. Striped bars indicate resistant female × susceptible male. Sample size for each strain or single-pair family = 48.

After 10 generations of selection with Cry1Ac in diet, results of bioassays with progeny from single-pair crosses demonstrated autosomal, dominant resistance to a diagnostic concentration of Cry1Ac in AY2 and QX7 (Fig. 2). For both strains, inheritance of resistance was autosomal, with essentially identical survival for the two reciprocal crosses for each strain (e.g., 96% for eight families from AY2♂ × SCD♀ and 97% for five families from SCD♂ × AY2♀, Fig. 2B). For the 13 single-pair families generated by reciprocal crosses between AY2 and SCD, survival at the diagnostic concentration ranged from 88 to 100% (mean = 96%, *h *=* *1.0, Fig. 2B). For the 20 single-pair F_1_ families derived from reciprocal crosses between QX7 and SCD, survival at the diagnostic concentration ranged from 88% to 98% for 18 families (mean = 93%, *h *=* *1.0, Fig. 2C) and was 48% for both of the other families. In the 18 families with 88% to 98% survival, we infer that the resistant parents from QX7 were homozygous for a dominant resistance allele (*RR*). The simplest explanation for the 48% survival in the two other families is that the resistant parents from QX7 were heterozygotes, with one copy of a dominant resistant allele (*R*) and one copy of a recessive susceptible allele (*s*), which would yield an expected 1:1 ratio of *Rs:ss* and 50% survival in the F_1_ progeny. From these results, we estimate that in QX7, after 10 successive generations of selection, the frequency was 0.9 (18/20) for *RR* and 0.1 (2/20) for *Rs*.

In contrast to the results with AY2 and QX7, survival at the diagnostic concentration of Cry1Ac was 0% for F_1_ progeny from both reciprocal crosses between resistant strain SCD-r1 and SCD (*h *=* *0, Table S1). These data show autosomal, recessive inheritance of resistance to Cry1Ac in SCD-r1, confirming previous results with this strain (Yang et al. 2009; Zhang et al. 2012b).

#### Dominance at different concentrations of Cry1Ac

**Figure 3:**
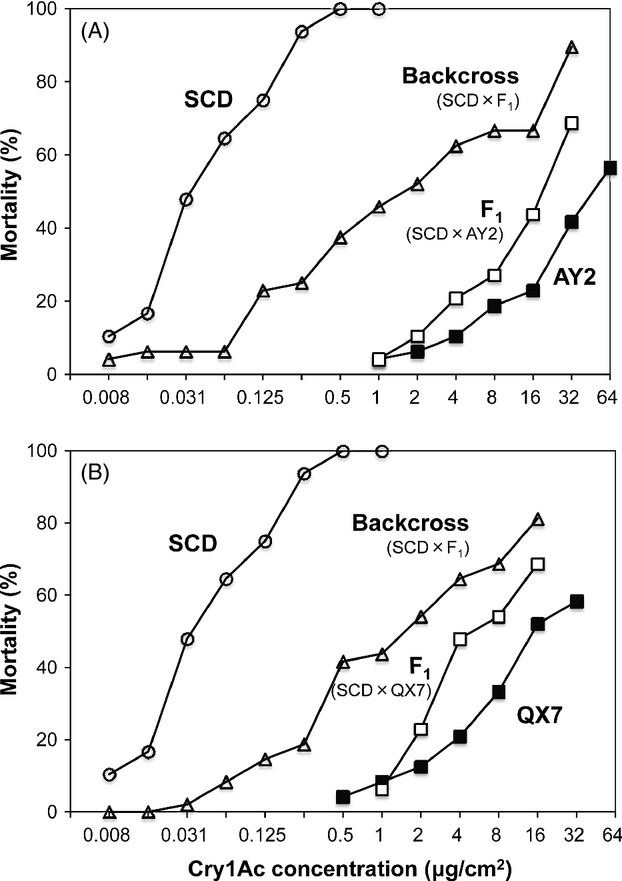
Responses to Cry1Ac of *H. armigera* larvae from a susceptible strain (SCD), resistant strains (AY2 and QX7), F_1_ progeny (resistant × SCD), and backcross progeny (F_1_ × SCD).

Responses of progeny from mass crosses to a series of concentrations of Cry1Ac in diet confirmed nonrecessive inheritance of resistance in AY2 and QX7 (Table 1, Figs 3 and S1). Based on the LC_50_ values (Table 1), *h* was 0.85 for AY2 and 0.83 for QX7. Similar to the results with single-pair families (Fig. 2), results with progeny from mass crosses show *h* was close to 1.0 at the diagnostic concentration (0.96 for AY2 and 0.98 for QX7) (Fig. S1). However, for both strains, dominance decreased as concentration increased, with the lowest values of *h* seen at the highest concentration tested against each strain (0.51 at 32 μg Cry1Ac/cm^2^ diet for AY2 and 0.62 at 16 μg Cry1Ac/cm^2^ diet for QX7, Fig. S1).

#### Number of loci conferring resistance to Cry1Ac in AY2 and QX7

As noted above, the single-pair F_1_ family data from QX7 fit a one-locus mode of resistance (Fig. 2C). In addition, based on three ways of analyzing data from backcrosses, the results are consistent with resistance conferred primarily by allelic variation at one locus in each strain (Table 1, Fig. 2, and Tables S2 and S3). In direct tests of a one-locus model, observed mortality did not differ significantly from expected mortality in progeny from a backcross between F_1_ and SCD for each of 11 comparisons (six concentrations for AY2 and five concentrations for QX7, Fisher's exact test, *P* > 0.25 in each comparison, Table S2). With indirect tests, observed mortality in backcrosses was generally similar to expected mortality from models with one, two, five, or ten loci (Table S3). Calculation of the minimum number of independently segregating loci with equal and additive contributions to resistance using data from strains, F_1_, and backcrosses (Lande 1981; Tabashnik et al. 1992, 2002) yielded an estimate of 0.68 for AY2 and 0.68 for QX7, which suggests resistance was conferred primarily by one locus in each strain. Although all of the results are consistent with a major locus conferring resistance in each strain, we cannot exclude the possibility of contributions from other loci.

#### Bt cotton leaves containing Cry1Ac

**Figure 4:**
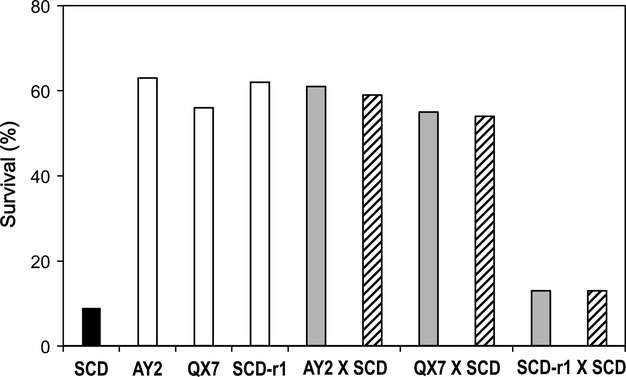
Survival on Bt cotton leaves of *H. armigera* larvae from a susceptible strain (SCD, black bar), three resistant strains (AY2, QX7 and SCD-r1, white bars), and the F_1_ progeny from crosses between each resistant strain and the susceptible strain (gray bars: resistant male × susceptible female, striped bars: resistant female × susceptible male).

Similar to the results for bioassays with a diagnostic concentration of Cry1Ac in diet (Fig. 2), inheritance of resistance to Bt cotton leaves producing Cry1Ac was dominant in AY2 (*h *=* *0.94) and QX7 (*h *=* *0.97), but recessive in SCD-r1 (*h *=* *0.08) (Fig. 4). Also consistent with results from the diet bioassays, inheritance of resistance to Bt cotton leaves was autosomal in all three resistant strains (Table S4). In the bioassays evaluating resistance to Bt cotton, we tested the parent resistant strains, the susceptible strain (SCD), and the F_1_ progeny from mass crosses between each resistant strain and the susceptible strain using leaves collected from field-grown Bt cotton plants (GK19) that produced Cry1Ac. The concentration of Cry1Ac in a subset of the field-collected leaves was 0.43 ± 0.05 μg Cry1Ac/gram leaf fresh weight, which is within the previously reported range of concentrations for leaves of field-grown GK19 cotton plants (Wan et al. 2005).

For AY2, QX7, and their F_1_ progeny from crosses with the susceptible strain, mean survival was lower on Bt cotton leaves (58%) than on diet with a diagnostic concentration of Cry1Ac (94%) (Figs 2 and 4, paired *t*-test, df = 5, *t* = 23, *P* < 0.001). Survival of SCD-r1 was similarly lower on Bt cotton leaves (62%) than on diet with a diagnostic concentration of Cry1Ac (94%) (Fisher's exact test, *P* < 0.001). However, survival was not lower on cotton leaves than on diet with a diagnostic concentration of Cry1Ac for either SCD (9% on leaves vs 0% on diet) or the F_1_ progeny from SCD-r1 × SCD (13% on leaves vs 0% on diet) (Figs 2 and 4).

### Cross-resistance to Cry1Aa, Cry1Ab, and Cry2Ab

For both AY2 and QX7, cross-resistance was highest to Cry1Aa (>260- and 100-fold, respectively), intermediate to Cry1Ab (69- and 34-fold, respectively), and lowest to Cry2Ab (5.9- and 4.2-fold, respectively) (Table 2).

**Table 2:** Cross-resistance to Cry1Aa, Cry1Ab, and Cry2Ab of Cry1Ac-selected strains (AY2 and QX7) relative to a susceptible strain (SCD) of *H. armigera*.

Strain	Bt toxin	LC_50_ (95% FL)*	Slope ± SE†	*n*	Resistance ratio‡
AY2	Cry1Aa	>80§	NA¶	432	>260
	Cry1Ab	25.7 (14–90)	1.3 ± 0.2	336	69
	Cry2Ab	0.338 (0.22–0.55)	1.1 ± 0.1	384	5.9
QX7	Cry1Aa	31.6 (18–120)	1.4 ± 0.2	240	100
	Cry1Ab	12.7 (7.1–38)	1.1 ± 0.1	288	34
	Cry2Ab	0.241 (0.20–0.30)	1.6 ± 0.1	432	4.2
SCD	Cry1Aa	0.313 (0.25–0.41)	1.6 ± 0.2	312	1.0
	Cry1Ab	0.373 (0.24–0.67)	1.7 ± 0.2	336	1.0
	Cry2Ab	0.0575 (0.043–0.074)	1.5 ± 0.2	336	1.0

* Concentration of toxin (μg/cm^2^) killing 50% of larvae and its 95% fiducial limits.

† Slope of the concentration–mortality line and its standard error.

‡ LC_50_ of a toxin for a strain divided by LC_50_ of the same toxin for susceptible strain SCD.

§ Mortality was 17% at 80 μg Cry1Aa/cm^2^ diet, the highest concentration tested.

¶ Not available.

## Discussion

The results from this study are consistent with previous studies reporting evidence of field-evolved resistance of *H. armigera* to Cry1Ac in northern China (Li et al. 2007; Liu et al. 2010; Zhang et al. 2011, 2012a). Survival at the diagnostic concentration of Cry1Ac was significantly higher in 2011 (this study) for Anyang (2.3%) and Qiuxian (1.4%) from northern China compared with two previously tested populations from northwestern China that had limited exposure to Bt cotton (0%, 0 of 1296, Zhang et al. 2011) (Fisher's exact test, *P* < 0.0001 for Anyang and Qiuxian tested separately). For Anyang, survival at the diagnostic concentration in 2011 was significantly higher than in 2005 (1.2%, 123 of 9984, Yang et al. 2007) (chi-squared = 10.7, df = 1, *P* = 0.001), but did not differ significantly from 2010 (2.6%, 33 of 1248, Zhang et al. 2011) (Fisher's exact test, *P* = 0.62). For Qiuxian, survival at the diagnostic concentration increased significantly from 2010 (0.2%, 2 of 888, Zhang et al. 2011) to 2011 (Fisher's exact test, *P* = 0.003).

The two resistant strains of *H. armigera* from northern China analyzed here, AY2 from Anyang and QX7 from Qiuxian, had dominant resistance to a diagnostic concentration of Cry1Ac in diet (*h *=* *1.0 for both strains) and dominant resistance to leaves of Bt cotton containing Cry1Ac (*h *=* *0.94 and 0.97, respectively). Assuming that at least one dominant resistance allele occurred in each set of individuals sampled from the field during 2011 to start each strain, we estimate the minimum frequency of individuals carrying a dominant resistance allele was 0.0045 for Anyang (1 of 222 field-collected moths) and 0.0056 for Qiuxian (1 of 178 field-collected moths). Because the frequency of individuals with resistance to Cry1Ac conferred by all alleles in 2011 was 0.023 for Anyang and 0.014 for Qiuxian (Results), we estimate the minimum percentage of resistant individuals carrying the dominant resistance alleles detected here as 20% for Anyang (0.0045/0.023) and 40% for Qiuxian (0.0056/0.014).

AY2 and QX7 had resistance ratios for Cry1Ac of 1200 and 460, as well as cross-resistance to Cry1Aa (>260- and 100-fold, respectively), Cry1Ab (69- and 34-fold, respectively), and Cry2Ab (5.9- and 4.2-fold, respectively) (Tables 1 and 2). For two other strains of *H. armigera* with high levels of resistance to Cry1Ac based on cadherin mutations (540-fold for SCD-r1 and 140-fold for XJ-r15), the magnitude of cross-resistance was lower to each toxin (Yang et al. 2009; Zhang et al. 2012b). However, as seen with AY2 and QX7, cross-resistance of SCD-r1 and XJ-r15 was highest to Cry1Aa (41- and 27-fold, respectively), intermediate to Cry1Ab (31- and 6.3-fold), and lowest to Cry2Ab (1.2- and 1.4-fold) (Yang et al. 2009; Zhang et al. 2012b).

Among 26 Cry1Ac-resistant strains of *H. armigera* from Australia, China, India, and Pakistan (Table 3), AY2 and QX7 have the resistance traits that appear to be most difficult to suppress. The resistance ratios for these two strains are among the highest (median for 22 other strains = 120-fold, range = 13–5400). The dominance (*h*) of resistance to Cry1Ac for AY2 and QX7 either at a diagnostic concentration in diet or in Bt cotton leaves containing Cry1Ac is higher than any reported previously (range of *h* for other strains = 0–0.66). Based on LC_50_ values, *h* was 0.85 for AY2 and 0.83 for QX7, which is similar to the maximum reported for 13 other strains (median = 0.39, range = 0–0.85). In contrast to the significant cross-resistance to Cry2Ab in AY2 and QX7 (Table 2), cross-resistance to Cry2Ab or Cry2Aa was not significant for any of the seven previously analyzed Cry1Ac-resistant strains of *H. armigera* considered individually (Table 3). However, the cross-resistance ratio for Cry2Ab or Cry2Aa was greater than one in eight of nine Cry1Ac-resistant strains of *H. armigera* (Table 2, median = 1.4, range = 1.0–5.9). Overall, for these nine strains, selection with Cry1Ac significantly decreased susceptibility to the Cry2A toxins (signed-rank test, one-tailed *P* < 0.005). Moreover, including the data for strains AY2 and QX7 reported here and the 21 selection experiments with *H. armigera* and seven other species of lepidopteran pests reviewed by Brévault et al. (2013), the cross-resistance ratio between Cry1A and Cry2A toxins was greater than one in 20 of 23 cases (median = 1.6, range = 0.32–420), with significant cross-resistance detected when all of the data are considered collectively (signed-rank test, one-tailed *P* = 0.0003).

**Table 3:** Resistance to Cry1Ac and cross-resistance to Cry2Ab in Cry1Ac-selected strains of *H. armigera*.

Country (region*)	Location	Year†	Strain	RR‡	Dominance (*h*) §								Cry2Ab CRR‡‡	References
					Cry1Ac (DC¶)	Cry1Ac (LC_50_**)	Bt cotton††							
China (N)	Anyang	2009	AY9	88	0.00				Zhang et al. (2012a)
China (N)	Anyang	2009	AY16		0.00				Zhang et al. (2012a)
China (N)	Anyang	2009	AY27		0.00				Zhang et al. (2012a)
China (N)	Anyang	2009	AY148		0.00				Zhang et al. (2012a)
China (N)	Anyang	2009	AY440	47	0.04				Zhang et al. (2012a)
China (N)	Anyang	2009	AY335	89	0.13				Zhang et al. (2012a)
China (N)	Anyang	2009	AY-r15	82	0.33	0.63			Zhang et al. (2012b)
China (N)	Anyang	2009	AY423	660	0.64				Zhang et al. (2012a)
China (N)	Anyang	2009	AY441	95	0.66				Zhang et al. (2012a)
China (N)	Anyang	2011	AY2	1200	1.0	0.85	0.94	5.9	This paper
China (N)	Gaoyang§§	2001	SCD-r1	440		0.00		1.2¶¶	Yang et al. (2009)
China (N)	Gaoyang§§	2001	SCD-r1	540	0.00	0.04			Zhang et al. (2012b)
China (N)	Gaoyang	2001	GYBT	560		0.24		1.4§§	Xu et al. (2005)
China (N)	Langfang	2000	LFR_10_	250***				1.0***	Luo et al. (2007)
China (N)	Qiuxian	2011	QX7	460	1.0	0.83	0.97	4.2	This paper
China (N)	Xiajin	2009	XJ-r15	140	0.65	0.68		1.4	Zhang et al. (2012b)
China (N)	Xinxiang	1996	BtR	3000***		0.28		1.1***	Luo et al. (2007), ¶ Liang et al. (2008)
China (NW)	Shache	2010	SC23	39	0.00				Zhang et al. (2012a)
China (NW)	Shawan	2010	SW34	31	0.26				Zhang et al. (2012a)
Australia	Mixed†††		BX	260		0.39	0.00, 0.63‡‡‡	1.4	Akhurst et al. (2003), Bird and Akhurst (2004, 2005)
India	Akola		Cry1Ac- resistant	72				1.1	Rajagopal et al. (2009)
India	Gujarat	2002	Res-Bt	93		0.42	0.43		Kranthi et al. (2006)
India	Gujarat	2006	BH-R	230		0.85§§§			Nair et al. (2010)
India	Nagpur	2002	Res-Ac	210		0.56			Kranthi et al. (2006)
India	Punjab	2005	BM-R	72	0.00¶¶¶	0.31			Kaur and Dilawari (2011)
India	Tamil Nadu		BCR	13		0.37			Shanmugam et al. (2007)
Pakistan	Punjab	2010	Cry1Ac- SEL	5400****		0.59			Alvi et al. (2012)

* N indicates northern China; NW indicates areas of northwestern China with limited planting of Bt cotton.

† The year when insects were sampled from the field to start the strain.

‡ Resistance ratio, LC_50_ of Cry1Ac for the resistant strain divided by LC_50_ of Cry1Ac for a susceptible strain.

§ Inheritance was autosomal in all strains except BH-R; when *h* was reported for each reciprocal cross, the mean is shown.

¶ *h* calculated from survival at a diagnostic concentration (1 μg Cry1Ac/cm^2^ diet unless noted otherwise; see Methods).

** *h* calculated from LC_50_ values of Cry1Ac (see Methods).

†† *h* calculated from survival on Bt cotton leaves unless noted otherwise (see Methods).

‡‡ Cross-resistance ratio; LC_50_ of Cry2Ab for the resistant strain divided by LC_50_ of Cry2Ab for a susceptible strain.

§§ The *r*_1_ allele from GYBT was introduced by repeated crossing and selection into the susceptible SCD strain.

¶¶ Based on Cry2Aa, which is similar to Cry2Ab.

*** Based on concentration of toxin causing 50% weight loss (WLC_50_) of the resistant strain divided by WLC_50_ of a susceptible strain.

††† Created by pooling three strains, one from Queensland and two apparently from New South Wales.

‡‡‡ 0.00 on young, intact plants; 0.63 on older, intact plants with a lower concentration of Cry1Ac.

§§§ 0.97 for the cross with resistant females; 0.73 for the reciprocal cross (mean *h *=* *0.85).

¶¶¶ Diagnostic concentration of 1 microgram Cry1Ac/mL diet.

**** Relative to the LAB-PK strain that was lab-selected for increased susceptibility.

Under selection for resistance, allele frequency is expected to increase faster for dominant resistance alleles than for recessive resistance alleles (Carrière and Tabashnik 2001). However, if dominant fitness costs are associated with the dominant resistance alleles found here, such costs could substantially slow the increase in these dominant alleles (Carrière and Tabashnik 2001). Dominant fitness costs would be especially effective for delaying resistance in this case because a high proportion of the host plants of *H. armigera* in northern China are crops other than cotton that do not produce Bt toxins and thus may act as refuges (Wu and Guo 2005). For example, from 2000 to 2006, Bt cotton accounted for a mean of only 7.5% of the total area planted to host plants of *H. armigera* each year in northern China (Wu et al. 2008).

Although complete assessment of costs in AY2 and QX7 will require additional work, some evidence suggests that a dominant fitness cost occurs in these strains. Both AY2 and QX7 had completely dominant resistance to Bt toxin Cry1Ac (*h *=* *1.0) based on evaluations made after 10 successive generations of laboratory selection with Cry1Ac. However, for both strains, survival was only 33% for the single-pair F_2_ progeny produced from the initial matings between one resistant F_1_ male survivor from the diagnostic concentration test and one female from the susceptible strain SCD (Fig. 1). With the results from both strains pooled (*n* = 96), the 33% observed survival is lower than the 50% survival expected with completely dominant resistance (Fisher's exact test, *P* = 0.028), assuming that the resistant male was a heterozygote (*Rs*), so that each cross (*Rs *× *ss*) is expected to yield 50% *Rs* that survive and 50% *ss* that die. The lower than expected survival could have been caused by a dominant fitness cost that reduced the proportion of *Rs* individuals from the mating between the *Rs* male and the *ss* female that became second instars and were tested in the bioassays.

Mortality of both *Rs* and *ss* individuals caused by factors other than Cry1Ac also could have contributed to the lower than expected survival. Another possibility is that dominance increased during the subsequent 10 generations of selection, which could have been mediated by modifiers at one or more loci other than the primary resistance locus or by replacement of the initial resistance allele by a more dominant resistance allele at the same locus. However, genetic variation was limited within strains because each strain was started with a single resistant male and a single female from a susceptible laboratory strain. In a previous study with the laboratory-selected BtR strain of *H. armigera*, Liang et al. (2008) reported a slight decrease in dominance as resistance increased during 87 generations of selection.

Whereas survival of the susceptible strain SCD was 9% in bioassays with leaves from China's popular GK19 variety of Bt cotton (Fig. 2 and Table S4), field data from 2001 and 2002 show that survival of larvae from susceptible populations of *H. armigera* on GK19 cotton was 8.2–18% (Wan et al. 2005). Thus, survival of the SCD strain in bioassays with GK19 cotton leaves was within the range of survival of susceptible field populations on GK19 plants in the field, which suggests that results from this bioassay are relevant to the field. Our leaf bioassays lasted only 5 days, which could boost survival relative to survival for longer periods required for complete larval development in the field. On the other hand, *H. armigera* larvae in the field eat a variety of plant parts, some of which have a much lower concentration of Cry1Ac than leaves, which could raise survival in the field relative to the leaf bioassays. In addition, in the field, the concentration of Cry1Ac declines in Bt cotton plants as they age (Wan et al. 2005), which increases survival of *H. armigera* larvae (Bird and Akhurst 2004, 2005). In greenhouse experiments with 15-week-old Bt cotton plants producing Cry1Ac, survival from neonate to adult was 62% for an *H. armigera* strain with a Cry1Ac resistance ratio of 97–440, 0% for a susceptible strain, and 39% for the F_1_ progeny of the resistant and susceptible strain, which had a Cry1Ac resistance ratio of 2–4 (Bird and Akhurst 2004, 2005). Similar to previous results with the Cry1Ac-selected Res-Bt strain of *H. armigera* from India (Kranthi et al. 2006) (Table 3), the results here with AY2 and QX7 show that dominance of resistance to Cry1Ac was similar whether measured in diet bioassays with Cry1Ac or in bioassays using Bt cotton leaves producing Cry1Ac (Figs 2 and 4, Table 3).

The high levels of resistance to Cry1Ac (1200- and 460-fold) and lower but significant cross-resistance to Cry2Ab (5.9- and 4.2-fold) of AY2 and QX7 raise concern about their potential resistance to two-toxin Bt cotton producing Cry1Ac and Cry2Ab. In bioassays with Bt cotton leaves containing Cry1Ac and Cry2Ab, survival was 13 times higher for the Cry1Ac-selected Res-Bt strain of *H. armigera* (32%) relative to a susceptible strain (2.4%) (Rajagopal et al. 2009), even though Res-Bt had only 72-fold resistance to Cry1Ac and no cross-resistance to Cry2Ab (Rajagopal et al. 2009). Similar results with the closely related pest species *Helicoverpa zea* show that survival from neonate to adult on Bt cotton producing Cry1Ac and Cry2Ab was 11 times higher for the Cry1Ac-selected GA-R strain (6.7%) relative to its unselected parent strain (0.6%), even though resistance of GA-R relative to GA was only 10-fold to Cry1Ac and twofold to Cry2Ab (Brévault et al. 2013).

While Cry1Ac is the only Bt toxin produced by transgenic cotton grown in China, two-toxin Bt cotton producing Cry1Ac and Cry2Ab has become the sole type of Bt cotton grown in Australia and the predominant type of Bt cotton grown in India and the United States (Tabashnik et al. 2013). An immediate switch in China to two-toxin Bt cotton producing Cry1Ac and Cry2Ab would probably slow the evolution of resistance to Bt cotton in *H. armigera* and in another major lepidopteran pest, *Pectinophora gossypiella* (Tabashnik et al. 2012). However, considering the increasing frequency of resistance of *H. armigera* in China to Cry1Ac and the concerns about an associated potential increase in survival on Bt cotton producing Cry1Ac and Cry2Ab described above, a shift to Bt cotton producing a toxin other than Cry1Ac or Cry2Ab could be particularly useful in China (Zhang et al. 2011).

Bt toxin Vip3Aa, which has no structural homology to Cry toxins (Estruch et al. 1996), is promising for controlling *H. armigera* populations (An et al. 2010; Mahon et al. 2012). Commercial varieties of three-toxin Bt cotton producing Vip3A, Cry1Ac, and Cry2Ab are under development, with availability in Australia and the United States expected in 2016 (Mahon et al. 2012). Susceptibility was not correlated between Cry1Ac and Vip3Aa within the Anci and Xiajin populations of *H. armigera* from northern China, and susceptibility was negatively associated between Cry1Ac and Vip3Aa across these two populations (An et al. 2010). In two Australian strains of *H. armigera* highly resistant to Vip3Aa, the mean LC_50_ of Cry1Ac was similar to that of a susceptible strain, while the mean LC_50_ of Cry2Ab was about fivefold lower than for a susceptible strain (Mahon et al. 2012). The frequency of recessive alleles conferring resistance to Vip3A was estimated as 0.008 in Australian populations of *H. armigera*, providing an indication of the potential for evolution of resistance to this toxin (Mahon et al. 2012). In addition to increasing the number and diversity of toxins in Bt cotton, integration of Bt cotton with other control tactics could help to delay the evolution of resistance and provide a more sustainable pest management system (Tabashnik et al. 2010).

## Acknowledegments

This work was funded by research grants from the Ministry of Agriculture of China (Grant 2013ZX08012-004), the National Natural Science Foundation of China (Grant 31071983), and the 111 program (Grant B07030).

## Data archiving statement

Raw data for this study are available as Supporting Information (Tables S1–S4) attached to the online version of the article.

## Supporting Information

Figure S1 Dominance (*h*) of resistance in AY2 and QX7 strains of *H. armigera* at different concentrations of Cry1Ac

**Table S1.**Dominance of resistance to a diagnostic concentration of Cry1Ac (1 μg Cry1Ac/cm^2^ diet) in three resistant strains of H. armigera (see Methods for details). **Table S2.** Direct tests show no significant difference between observed mortality and mortality expected from a one-locus model for backcross progeny tested against Cry1Ac in diet. **Table S3**. Indirect tests for fit between observed mortality and mortality expected from models with 1, 2, 5 or 10 loci for backcross progeny tested against Cry1Ac in diet. **Table S4**. Dominance of resistance to Bt cotton leaves (0.43 μg ± 0.05 μg Cry1Ac/g leaf fresh weight) in three resistant strains of *H. armigera* (see Methods for details).
