# Supplementary material for: Dominant resistance to Bt cotton and minor cross-resistance to Bt toxin Cry2Ab in cotton bollworm from China
Source: Evol Appl. 2013 Sep 17;6(8):1222–35. doi: 10.1111/eva.12099 (PMC3901552; doi:10.1111/eva.12099)
Supplement: Supplementary file 2 — Table S1.Dominance of resistance to a diagnostic concentration of Cry1Ac (1 μg Cry1Ac/cm2 diet) in three resistant strains of H. armigera (see Methods for details). Table S2. Direct tests show no significant difference between observed mortality and mortality expected from a one-locus model for backcross progeny tested against Cry1Ac in diet. Table S3. Indirect tests for fit between observed mortality and mortality expected from models with 1, 2, 5 or 10 loci for backcross progeny tested against Cry1Ac in diet. Table S4. Dominance of resistance to Bt cotton leaves (0.43 μg ± 0.05 μg Cry1Ac/g leaf fresh weight) in three resistant strains of H. armigera (see Methods for details). [file eva0006-1222-sd2.docx]

**Table S1.** Dominance of resistance to a diagnostic concentration of Cry1Ac (1 μg Cry1Ac/cm^2^ diet) in three resistant strains of *H. armigera* (see Methods for details).

| Resistant  strain* | Survival (%)† | | | *h*‡ |
| --- | --- | --- | --- | --- |
|  | Resistant ×  resistant | Resistant♂ ×  SCD♀ | Resistant♀ ×  SCD♂ |  |
| AY2 | 92 | 96 (8) | 97 (5) | 1.0 |
| QX7 | 92 | 94 (8)§ | 93 (10)§ | 1.0 |
| SCD-r1 | 94 | 0 | 0 | 0.0 |

*AY2 and QX7 were derived from the field in 2011; resistance in the lab-selected strain SCD-r1 is conferred by a cadherin mutation.

†Survival was 0% for the susceptible laboratory strain SCD. We tested progeny from single-pair crosses between AY2 and SCD as well as between QX7 and SCD. The values in parentheses indicate the number of single-pair families tested. To obtain all other progeny tested, we used mass crosses with at least 30 adults of each sex. We tested 48 larvae from each strain, mass cross, or single-pair family. For example, we tested a total of 480 larvae from the 10 single-pair crosses between QX7 females and SCD males and a total of 96 larvae from the two reciprocal mass crosses between SCD-r1 and SCD.

‡Values of *h* vary from 0 for completely recessive resistance to 1 for completely dominant resistance.

§Excludes one single-pair family with 48% survival from each reciprocal cross between QX7 and SCD (see Fig. 2). We infer that the parent from QX7 in each of the two single-pair crosses with 48% survival in the progeny was a heterozygote (*Rs*) and thus we did not include these families in calculating survival or *h.*

**Table S2.** Direct tests show no significant difference between observed mortality and mortality expected from a one-locus model for backcross progeny tested against Cry1Ac in diet.

| Backcross* | Concn.† | Observed | | Expected‡ | | *P*§ |
| --- | --- | --- | --- | --- | --- | --- |
|  |  | Live | Dead | Live | Dead |  |
| (AY2 × SCD) × SCD | 1 | 26 | 22 | 46 | 50 | 0.60 |
|  | 2 | 23 | 25 | 43 | 53 | 0.73 |
|  | 4 | 18 | 30 | 38 | 58 | 0.86 |
|  | 8 | 16 | 32 | 35 | 61 | 0.85 |
|  | 16 | 16 | 32 | 27 | 69 | 0.56 |
|  | 32 | 5 | 43 | 15 | 81 | 0.45 |
| (QX7 × SCD) × SCD | 1 | 27 | 21 | 45 | 51 | 0.38 |
|  | 2 | 22 | 26 | 37 | 59 | 0.47 |
|  | 4 | 17 | 31 | 25 | 71 | 0.25 |
|  | 8 | 15 | 33 | 22 | 74 | 0.31 |
|  | 16 | 9 | 39 | 15 | 81 | 0.64 |

*Results with F_1_ progeny show autosomal inheritance of resistance to Cry1Ac (i.e., no sex linkage and no maternal effects) (Fig. 2). To obtain backcross progeny, we first generated F_1_ progeny by crossing resistant males with susceptible females, then we crossed F_1_ males with susceptible females. We used 30 males and 30 females for each cross.

†μg Cry1Ac per cm^2^ diet.

‡Calculated based on a one-locus model (Tabashnik et al. 1992) assuming F_1_ progeny were *Rs* and SCD individuals were *ss*, yielding 50% *Rs* and 50% *ss* in the progeny from the backcross between F_1_ (AY2 × SCD or QX7 × SCD) and SCD.

§Probability that the difference between the proportion of observed and expected mortality occurred at random (Fisher's exact test, http://graphpad.com/quickcalcs/contingency1.cfm).

**Table S3.** Indirect tests for fit between observed mortality and mortality expected from models with 1, 2, 5 or 10 loci for backcross progeny tested against Cry1Ac in diet.

| Backcross* | No. of loci in genetic model | Mean absolute  difference (%)† | Concentrations with a  significant difference between obs. and exp.‡ |
| --- | --- | --- | --- |
| (AY2 × SCD) × SCD | 1 | 6.8 | 0 |
|  | 2 | 3.8 | 0 |
|  | 5 | 5.1 | 0 |
|  | 10 | 8.4 | 2§ |
| (QX7 × SCD) × SCD | 1 | 6.5 | 0 |
|  | 2 | 2.6 | 0 |
|  | 5 | 3.9 | 0 |
|  | 10 | 6.5 | 0 |

*For both backcrosses, we tested backcross progeny at the following 11 concentrations: 0.0156, 0.031, 0.0625, 0.125, 0.25, 0.5, 1, 2, 4, 8, and 16 μg Cry1Ac per cm^2^ diet. For the backcross involving AY2, we tested two additional concentrations: 0.0078 and 32 μg Cry1Ac per cm^2^ diet. See Supplementary Table 2 for additional details about the backcross methods.

†Mean of the absolute difference between observed and expected mortality. Expected mortality and this parameter were calculated as described by Tabashnik et al. 1992. In models with 2, 5 and 10 loci, effects of resistance alleles at each locus were equal and additive. With the exception of the 10-locus model for the backcross involving AY2, the mean absolute difference was <7%, which indicates a good fit between observed and expected mortality (Tabashnik et al. 1992, 2002).

‡We used Fisher's exact test (http://graphpad.com/quickcalcs/contingency1.cfm) to determine if a significant difference (*P* < 0.05) occurred between observed and expected mortality at each concentration tested. With the exception of the 10-locus model for the backcross involving AY2, no significant difference was detected between observed and expected mortality for any of the concentrations tested.

§At 0.125 μg Cry1Ac per cm^2^ diet, observed mortality (23%) was significantly higher than expected mortality (6.7%) (P = 0.04) and at 16 μg Cry1Ac per cm^2^ diet, observed mortality (67%) was significantly lower than expected mortality (88%) (P = 0.02).

**Table S4.** Dominance of resistance to Bt cotton leaves (0.43 μg ± 0.05 μg Cry1Ac per g leaf fresh weight) in three resistant strains of *H. armigera* (see Methods for details).

| Resistant  strain* | Survival (%)† | | | *h*‡ |
| --- | --- | --- | --- | --- |
|  | Resistant × Resistant | Resistant♂ × SCD♀ | Resistant♀ × SCD♂ |  |
| AY2 | 63 | 61 | 59 | 0.94 |
| QX7 | 56 | 55 | 54 | 0.97 |
| SCD-r1 | 62 | 13 | 13 | 0.08 |

*AY2 and QX7 were derived from the field in 2011; resistance in the lab-selected strain SCD-r1 is conferred by a cadherin mutation.

†Survival was 9% for the susceptible laboratory strain SCD. We tested progeny from mass crosses (with at least 30 adults of each sex). From each mass cross, we tested 30 replicates of 5 larvae each. For example, we tested a total of 150 larvae from AY2 and a total of 300 larvae from the two reciprocal mass crosses between AY2 and SCD.

‡Values of *h* vary from 0 for completely recessive resistance to 1 for completely dominant resistance.
